# Supplementary material for: Dynamic Evolution of Aquaculture along the Bohai Sea Coastline and Implications for Eco-Coastal Vegetation Restoration Based on Remote Sensing
Source: Plants (Basel). 2024 Jan 6;13(2):160. doi: 10.3390/plants13020160 (PMC10818457; doi:10.3390/plants13020160)
Supplement: Supplementary file 1 [file plants-13-00160-s001.zip › plants-2696748-supplementary.pdf]

The data identification of the Landsat images

1984

LT05_L1TP_118032_19840914_20170220_01_T1

LT05_L1TP_119032_19840905_20170220_01_T1

LT05_L1TP_119033_19840905_20170220_01_T1

LT05_L1TP_119034_19840905_20170220_01_T1

LT05_L1TP_120032_19840827_20170220_01_T1

LT05_L1TP_120033_19840827_20170220_01_T1

LT05_L1TP_120034_19840827_20170220_01_T1

LT05_L1TP_121032_19840903_20170220_01_T1

LT05_L1TP_121033_19840919_20170220_01_T1

LT05_L1TP_121034_19840717_20170220_01_T1

LT05_L1TP_122033_19840910_20170220_01_T1

LT05_L1TP_122034_19840910_20170220_01_T1

1987

LT05_L1TP_118032_19870603_20170212_01_T1

LT05_L1TP_119032_19870626_20170212_01_T1

LT05_L1TP_119033_19870626_20170212_01_T1

LT05_L1TP_119034_19870930_20170418_01_T1

LT05_L1TP_120032_19871007_20170211_01_T1

LT05_L1TP_120033_19870921_20170211_01_T1

LT05_L1TP_120034_19870921_20170211_01_T1

LT05_L1TP_121032_19870912_20170418_01_T1

LT05_L1TP_121033_19870912_20170418_01_T1

LT05_L1TP_121034_19870608_20170212_01_T1

LT05_L1TP_122033_19870919_20170211_01_T1

LT05_L1TP_122034_19870919_20170211_01_T1

1992

LT05_L1TP_118032_19920920_20170122_01_T1

LT05_L1TP_119032_19921013_20170122_01_T1

LT05_L1TP_119033_19920607_20170123_01_T1

LT05_L1TP_119034_19920927_20170214_01_T1

LT05_L1TP_120032_19920817_20170122_01_T1

LT05_L1TP_120033_19920614_20170124_01_T1

LT05_L1TP_120034_19920801_20170214_01_T1

LT05_L1TP_121032_19920909_20170214_01_T1

LT05_L1TP_121033_19920925_20170121_01_T1

LT05_L1TP_121034_19920909_20170214_01_T1

LT05_L1TP_122033_19920527_20170122_01_T1

LT05_L1TP_122034_19921018_20170122_01_T1

1997

LT05_L2SP_118032_19970614_20200910_02_T1

LT05_L2SP_119032_19970707_20200910_02_T1

LT05_L2SP_119033_19970707_20200910_02_T1

LT05_L2SP_119034_19970621_20200910_02_T1

LT05_L2SP_120034_19971018_20200909_02_T1

LT05_L2SP_120032_19971018_20200909_02_T1

LT05_L2SP_120033_19971018_20200910_02_T1

LT05_L2SP_121032_19971009_20200909_02_T1

LT05_L2SP_121033_19971009_20200909_02_T1

LT05_L2SP_121034_19970923_20200909_02_T1

LT05_L2SP_122033_19970930_20200910_02_T1

LT05_L2SP_122034_19970813_20200910_02_T1

2002

LE07_L2SP_118032_20020519_20200917_02_T1

LE07_L2SP_119032_20020830_20200916_02_T1

LE07_L2SP_119033_20020830_20200916_02_T1

LE07_L2SP_119034_20021001_20200916_02_T1

LE07_L2SP_120034_20020906_20200916_02_T1

LE07_L2SP_120032_20020922_20200916_02_T1

LE07_L2SP_120033_20020922_20200916_02_T1

LE07_L2SP_121032_20021015_20200916_02_T1

LE07_L2SP_121033_20021015_20200916_02_T1

LE07_L2SP_121034_20021015_20200916_02_T1

LE07_L2SP_122033_20021006_20200916_02_T1

LE07_L2SP_122034_20020531_20200917_02_T1

2007

LT05_L1TP_118032_20070930_20161110_01_T1

LT05_L1TP_119032_20070921_20161112_01_T1

LT05_L1TP_119033_20070905_20161111_01_T1

LT05_L1TP_119034_20070921_20161112_01_T1

LT05_L1TP_120034_20070421_20161115_01_T1

LT05_L1TP_120032_20070928_20161110_01_T1

LT05_L1TP_120033_20070928_20161110_01_T1

LT05_L1TP_121032_20070615_20161113_01_T1

LT05_L1TP_121033_20070514_20161115_01_T1

LT05_L1TP_121034_20070514_20161115_01_T1

LT05_L1TP_122033_20070403_20161116_01_T1

LT05_L2SP_122034_20070809_20200830_02_T1

2012

LC08_L1TP_118032_20131016_20170429_01_T1

LC08_L1TP_119032_20130516_20170504_01_T1

LC08_L1TP_119033_20130516_20170504_01_T1

LC08_L1TP_119034_20130820_20170502_01_T1

LC08_L1TP_120032_20131030_20170429_01_T1

LC08_L1TP_120033_20130523_20170504_01_T1

LC08_L1TP_120034_20130523_20170504_01_T1

LC08_L1TP_121032_20130530_20170504_01_T1

LC08_L1TP_121033_20131005_20170429_01_T1

LC08_L1TP_121034_20131005_20170429_01_T1

LC08_L1TP_122033_20130926_20170502_01_T1

LC08_L2SP_122034_20131012_20200912_02_T1

2017

LC08_L2SP_118032_20170605_20200903_02_T1

LC08_L2SP_119032_20171002_20200903_02_T1

LC08_L2SP_119033_20171002_20200903_02_T1

LC08_L1TP_119034_20171002_20200903_02_T1

LC08_L2SP_120032_20170923_20200903_02_T1

LC08_L2SP_120033_20170923_20200903_02_T1

LC08_L1TP_120034_20170923_20171012_01_T1

LC08_L1TP_121032_20171016_20171024_01_T1

LC08_L1TP_121033_20170712_20170726_01_T1

LC08_L1TP_121034_20170930_20171013_01_T1

LC08_L1TP_122033_20171023_20171107_01_T1

LC08_L2SP_122034_20171023_20200902_02_T1

2022

LC08_L2SP_118032_20221025_20221107_02_T1

LC09_L2SP_119032_20220906_20230330_02_T1

LC08_L2SP_119033_20220930_20221005_02_T1

LC08_L2SP_119034_20220930_20221005_02_T1

LC08_L2SP_120032_20221007_20221013_02_T1

LC08_L2SP_120033_20221007_20221013_02_T1

LC08_L2SP_120034_20221023_20221101_02_T1

LC09_L2SP_121032_20221022_20230325_02_T1

LC08_L2SP_121033_20220928_20221004_02_T1

LC08_L2SP_121034_20220928_20221004_02_T1

LC08_L2SP_122033_20220919_20220928_02_T1

LC08_L2SP_122034_20221005_20221012_02_T1
